# Survey of Microbial Diversity in Flood Areas during Thailand 2011 Flood Crisis Using High-Throughput Tagged Amplicon Pyrosequencing

**DOI:** 10.1371/journal.pone.0128043

**Published:** 2015-05-28

**Authors:** Wuttichai Mhuantong, Sarunyou Wongwilaiwalin, Thanaporn Laothanachareon, Lily Eurwilaichitr, Sithichoke Tangphatsornruang, Benjaporn Boonchayaanant, Tawan Limpiyakorn, Kobchai Pattaragulwanit, Thantip Punmatharith, John McEvoy, Eakalak Khan, Manaskorn Rachakornkij, Verawat Champreda

**Affiliations:** 1 Enzyme Technology Laboratory, National Center for Genetic Engineering and Biotechnology, 113 Thailand Science Park, Phahonyothin Road, Khlong Luang, Pathum Thani 12120, Thailand; 2 Genome Institute, National Center for Genetic Engineering and Biotechnology, Thailand Science Park, Khlong Luang, Pathum Thani 12120, Thailand; 3 Department of Environmental Engineering, Faculty of Engineering, Chulalongkorn University, Phayatai, Bangkok 10330, Thailand; 4 Center of Excellence on Hazardous Substance Management (HSM), Chulalongkorn University, Phayatai, Bangkok 10330, Thailand; 5 Department of Microbiology, Faculty of Science, Chulalongkorn University, Phayatai, Bangkok 10330, Thailand; 6 Department of Veterinary and Microbiological Sciences, North Dakota State University, Fargo, ND 58108, United States of America; 7 Department of Civil and Environmental Engineering, North Dakota State University, Fargo, ND 58108, United States of America; CAS, CHINA

## Abstract

The Thailand flood crisis in 2011 was one of the largest recorded floods in modern history, causing enormous damage to the economy and ecological habitats of the country. In this study, bacterial and fungal diversity in sediments and waters collected from ten flood areas in Bangkok and its suburbs, covering residential and agricultural areas, were analyzed using high-throughput 454 pyrosequencing of 16S rRNA gene and internal transcribed spacer sequences. Analysis of microbial community showed differences in taxa distribution in water and sediment with variations in the diversity of saprophytic microbes and sulfate/nitrate reducers among sampling locations, suggesting differences in microbial activity in the habitats. Overall, Proteobacteria represented a major bacterial group in waters, while this group co-existed with Firmicutes, Bacteroidetes, and Actinobacteria in sediments. *Anaeromyxobacter*, *Steroidobacter*, and *Geobacter* were the dominant bacterial genera in sediments, while *Sulfuricurvum*, *Thiovirga*, and *Hydrogenophaga* predominated in waters. For fungi in sediments, Ascomycota, Glomeromycota, and Basidiomycota, particularly in genera *Philipsia*, *Rozella*, and *Acaulospora*, were most frequently detected. Chytridiomycota and Ascomycota were the major fungal phyla, and *Rhizophlyctis* and *Mortierella* were the most frequently detected fungal genera in water. Diversity of sulfate-reducing bacteria, related to odor problems, was further investigated using analysis of the *dsr*B gene which indicated the presence of sulfate-reducing bacteria of families Desulfobacteraceae, Desulfobulbaceae, Syntrobacteraceae, and Desulfoarculaceae in the flood sediments. The work provides an insight into the diversity and function of microbes related to biological processes in flood areas.

## Introduction

In 2011, Thailand witnessed the most enormous flood in its history, which devastated infrastructure, agriculture, industry, and society as a whole. The flood swept from the north of Chao Phraya River basin through Central Thailand during July 2011 to January 2012, submerging 60,000 square kilometers of land. The flood is ranked as one of the world’s costliest natural disasters, affecting more than 13 million people, with estimated economic damages reaching 45 billion US$, and resulting shortages in global industrial supply chains (http://www.worldbank.org). The tropical savanna climate of Thailand makes the country prone to flooding during the monsoon season. With the impact of La Nina causing increasing tropical storms and annual rainfall, large-scale flooding is expected.

In terms of ecology, flash flooding causes immense damage to ecological niches in flooded areas, resulting in changes in microbial communities. Microbial diversity in floodwater is related to decomposition of organic matters and other biochemical activities in water, such as photosynthesis, recycling of nutrients, and sulfate/nitrate reduction, which are directly related to water properties [1]. Compared to diversity in aquatic and marine environments, the microbial community in floodwater is temporally variable, but has a higher impact on human habitat and public health issues [2, 3]. This includes contamination of floodwater to water sources for human consumption, which can cause widespread health public problems in the area [4]. Few studies have examined microbial diversity during flooding, and these have used conventional, culture-dependent isolation techniques which thus generated only a limited insight on the microbial diversity in flood areas [3].

High-throughput sequencing is a powerful tool for the study of complex community structure and functions of microbial assemblages in environments. Tagged amplicon pyrosequencing or bacterial tag encoded FLX amplicon pyrosequencing (bTEFAB) has been introduced for high-throughput sequencing of specific target genes, particularly the 16S rRNA gene as a marker for biodiversity [5, 6]. This culture-independent technique also has been used to explore microbial community structure in aquatic and marine environments, such as the comparison of bacterial diversity in sediments from fresh water, intertidal wetland, and marine sediments [7], and profiling of archaea, bacteria, and small eukaryotes in the coastal areas [8].

In this study, bacterial and fungal diversity in sediment and floodwater in different locations covering agricultural and residential areas was investigated using tagged amplicon pyrosequencing of the 16S rRNA gene and internal transcribed spacer (ITS). Comparative microbial profiles in different areas were analyzed for taxon predominance in these unique ecological niches. Diversity of sulfate-reducing bacteria known to be involved in odor generation in floodwater was further investigated by analyzing the specific marker gene in the dissimilatory sulfate-reduction pathway. The work represents one of the first extensive surveys on microbial diversity in flooding areas using high-throughput sequencing, which gives insights to microbial functions and biological processes during flooding.

## Materials and Methods

### Sample collection and physicochemical analysis

**Fig 1:**
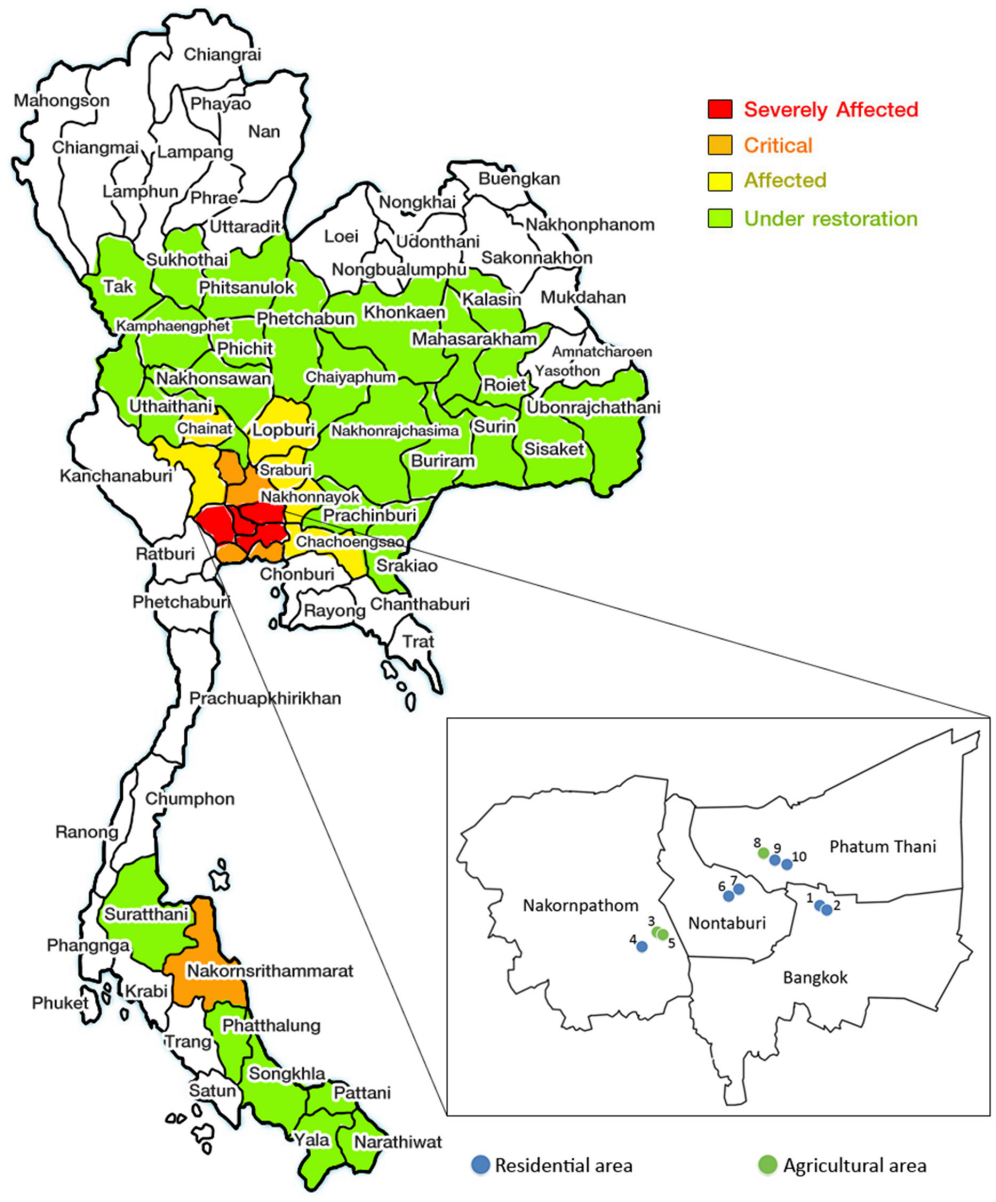
Map of sample collection sites. The sampling areas are in Bangkok, Pathumthani, Nonthaburi, and Nakornpathom provinces.

Sediment (S) and floodwater (W) samples were collected in December 2011 from 10 locations in Bangkok and suburban areas in Nakornpathom, Nontaburi, and Phatum Thani provinces (Fig 1). The sampling sites covered agricultural and residential areas (5–150 cm water depth). These locations are in public areas and need no permission for sample collection. The field studies did not involve disturbance of any endangered or protected species. The sediment samples were collected at 2.5–15 cm below the surface. Three samples were collected as field replicates per site studied. The replicate samples were pooled and thoroughly mixed. Physical and chemical characteristics of the samples (temperature, pH, dissolved oxygen (DO), oxidation-reduction potential (ORP), and conductivity) were analyzed using Standard Methods [9]. DO was measured on Dissolved Oxygen Portable Meter (Orion 3 star, Thermoscientific, USA), ORP was measured using a pH/ORP Waterproof portable detector (HI 98183, Hana Instrument, USA) and Conductivity was analyzed on Portable Conductivity Meter (HQ14d, Hach, USA). The samples were kept at 4°C during transportation. The sediment and water samples were processed and kept at -20°C before DNA extraction.

### DNA extraction

Environmental DNA for the sediment samples was extracted from 0.25 g of sample (oven dried basis) using a Power Soil DNA Extraction kit (MO-BIO Laboratories, Carlsbad, CA) according to a procedure recommended by the manufacturer. The water samples (500 mL) were filtered through a 0.2 μm pore-size membrane (Supor 200, Pall, Port Washington, NY) to remove the suspended solids and processed for environmental DNA extraction using a Power Water DNA Extraction kit (MO-BIO Laboratories). The integrity of the extracted DNA was confirmed by agarose gel electrophoresis. The pool of 3 independent purified metagenomic DNA samples from each sample was then processed for target gene amplification and pyrosequencing. The concentration of extracted DNA was quantified using a NanoDrop ND-1000 spectrophotometer (PeqlabBiotechnologie GmbH, Erlangen, Germany).

### Tagged 16S rRNA gene/ ITS amplicon sequencing

The purified metagenomic DNA (sample S1–S10 and W1–W10) was used as a template for amplification of the partial 16S rRNA gene using universal bacterial primers 454B338F (5’- ACTCCTACGGGAGGCAGCAG-3’) [10] and 454B786R (5’-CTACCAGGGTATCTAATC -3’) encompassing the 3 and 4 hypervariable regions in prokaryotic 16S rRNA gene [11] or ITS1F (5’-CTTGGTCATTTAGAGGAAGTAA-3’) [12] and ITS4 (5’-TCCTCCGCTTATTG ATATGC-3’) [13] primers for amplification of the ITS1 and ITS2 regions of the ITS sequence. The primers were attached with tagged barcode sequences for sample grouping [14]. Polymerase chain reactions were performed using DyNAzyme EXT DNA polymerase (Finnzyme, Espoo, Finland) on a MyCycler thermocycler (Bio-Rad, Hercules, CA) using pre-denaturation at 94°C for 3 min, and then 30 cycles of denaturation at 94°C for 1 min, annealing at 55°C for 1 min, and extension at 72°C for 1 min 45 s with a final prolonged extension at 72°C for 10 min. The purified amplicons were quantified using a NanoDrop ND-1000 spectrophotometer. The sequences were determined using on a 454-Life Sciences GS-FLX Genome Sequencer System (Roche, Branford, CT) following the recommended protocols by the manufacturer. The sequencing datasets were deposited in the NCBI Sequence Read Archive (SRA) under the accession number SRP041480.

### Data cleaning and diversity analyses

The raw sequence reads were initially classified into specific groups based on their tagged barcode sequences and then trimmed by removing their barcode and primer sequences at the 5’ end of reads. Chimera, hybrid sequences merged from multiple parent sequences due to the error of PCR amplification, were identified and removed by UCHIME [15] using referenced databases from SILVA [16] and UNITE [17] for bacterial 16S rRNA gene and fungal ITS, respectively. Sequences that were 100 bps or longer in length were selected for analysis and clustered into operational taxonomic units (OTUs) at sequence dissimilarity levels of 0.03, 0.05, and 0.15 by the furthest-neighbor method. In order to reduce the bias of unequal number of datasets, normalization was performed using subsampling method in MOTHUR before calculating the diversity index [18]. The Shannon-Weaver index was calculated for measuring and comparing the diversity among datasets. Meanwhile, the Chao1 richness estimator was calculated to estimate number of OTUs for each sample. Good's coverage was used as an estimator for sampling completeness by calculating from G = 1-(n/N), where n is the number of singleton phylotypes and N is the total number of sequences in the sample.

For diversity analysis, the taxonomic classification of bacteria was assigned based on the processed 16S rRNA gene sequences using a naïve Bayesian classifier (RDP classifier) [19] to determine approximate phylogeny with 80% confidence threshold. Identification at the species level for the purpose of this study is considered tentative. The fungal taxonomy was assigned by BLASTN against referenced ITS database [20] using an expected E-value cutoff of 1e-03. Statistical and pairwise analyses of microbial profiles were analyzed and visualized by STAMP (Statistical Analysis of Metagenomic Profiles) [21].

### Analysis of sulfate-reducing bacterial community using PCR-DGGE of *dsr*B genes

Sulfate-reducing bacterial communities in sediment and water samples were investigated using denaturing gradient gel electrophoresis (DGGE) of the sulfite reductase (*dsr*B gene) fragment. A fragment of *dsrB* gene was amplified from selected sediment and water DNA samples with primers DSRp2060F (5’-CAACATCGTYCAYACCCAGGG-3’) attached to a GC-clamp [22] and DSB4R (5’-GTGTAGCAGTTACCGCA-3’) [23]. Thermal cycling was carried out with an initial denaturation step of 94°C for 4 min, followed by 35 cycles of denaturation at 94°C for 1 min, annealing at 55°C for 1 min, and elongation at 72°C for 1 min with a final extension step at 72°C for 10 min. The *dsr*B gene profiles were analyzed by DGGE using the DCode system (Bio-Rad Laboratories, Hercules, CA) on a 8% polyacrylamide gel with 40–70% gradient of the urea formamide denaturant [24]. The electrophoresis was run for 15 h at 60°C and 120 V. The gel was stained with ethidium bromide for visualization. Prominent bands were excised and dissolved in 50 μl sterilized water. The DNA was recovered as used as a template for re-amplification of the gene. DNA sequences were analyzed by Macrogen (Seoul, South Korea). The sequences were analyzed for preliminary identity by the Advanced BLASTN Search program [25]. The analyzed sequences were aligned with sequences selected from Genbank to represent groups of known SRB using the ARB program package (Department of Microbiology, Technische Universitat Munchen, Munich, Germany; [http://www.arb-home.de]). The phylogenetic tree was constructed using the ARB program package. A neighbor-joining tree was constructed using almost full-length sequences of *dsr*AB genes of reference SRB. Then, our fragments of dsrB genes were added into the tree using parsimony method available in the package to avoid the change in tree topology when short sequences were used.

## Results

### Sample collection and characterization

Sediments (S) and floodwater samples (W) were collected from 10 selected locations in Bangkok and its suburbs, covering residential, agricultural, and industrial areas (Fig 1 and Table 1). The depth of water was between 5 cm and 1.5 m. The samples showed variation in physical and chemical characteristics, which reflected differences in their micro-environmental conditions for microbial growth. DO in the samples varied from 0.44–5.97 mg/L. The dissolved oxygen below 1 mg/L was observed in samples 1, 4 and 10, reflecting poor water quality and relatively microaerobic condition. The sample temperatures at the time of collection were in the range of 23.5–29.6°C. pH was in the neutral range and showed no substantial difference among the samples. Conductivity values, which reflect the amount of dissolved solids in water, were in the range of 223–798 μS/cm. Oxidation-reduction potentials (ORP) of the samples varied among the locations. In all samples, ORP values in the sediment were lower than those in the water, suggesting more reduced conditions were present in the sediment. For samples 1, 2, and 5, negative ORP values were observed in the sediment, reflecting anaerobic conditions at these locations.

**Table 1:** Locations of sample collections and physical/ chemical properties of the samples.

No.	GPS (UTM)	Source	Sample ID	Sampling depth(cm.)	Type	Water depth(cm.)	Temp (°C)	DO (mg/L)	pH	Conductivity (μS/cm)	ORP (mV)
1	X:0674835	sediment	BS1/FS1	2.5	Residential	-	-	-	6.9	628	-272.4
	Y:1537632	water	BW1/FW1	0.5	Residential	5	24.2	0.44	7.2	645	483.5
2	X:0674854	sediment	BS2/FS2	10	Residential	-	-	-	7.4	534	-137.5
	Y:1537564	water	BW2/FW2	2.5	Residential	20	25.4	4.93	6.9	545	215.2
3	X:0641277	sediment	BS3/FS3	15	Agricultural	-	-	-	6.8	403	30.6
	Y:1530581	water	BW3/FW3	5	Agricultural	120	24.9	2.58	6.9	401	56.4
4	X:0638142	sediment	BS4/FS4	5	Residential	-	-	-	7.2	359	124.3
	Y:1526684	water	BW4/FW4	2.5	Residential	15	29.6	0.91	7.2	358	159.8
5	X:0641230	sediment	BS5/FS5	15	Agricultural	-	-	-	6.5	374	-104.7
	Y:1530640	water	BW5/FW5	5	Agricultural	120	24.7	2.65	6.7	369	34.7
6	X:0653186	sediment	BS6/FS6	10	Residential	-	-	-	7.2	356	218.7
	Y:1538533	water	BW6/FW6	0.5	Residential	30	26.4	4.26	6.5	344	232.8
7	X:0654755	sediment	BS7/FS7	10	Residential	-	-	-	7.2	285	126.8
	Y:1539213	water	BW7/FW7	0.5	Residential	20	27.3	5.97	7.3	270	218.6
8	X:0656741	sediment	BS8/FS8	10	Agricultural	-	-	-	7.1	299	179.4
	Y:1551880	water	BW8/FW8	10	Agricultural	70	29.3	3.83	7.6	302	181.0
9	X:0654858	sediment	BS9/FS9	10	Residential	-	-	-	6.8	232	90.7
	Y:1552939	water	BW9/FW9	5	Residential	> 150	26.1	1.24	7.0	223	105.3
10	X:0653355	sediment	BS10/FS10	5	Residential	-	-	-	7.3	798	128.2
	Y:1552636	water	BW10/FW10	0.5	Residential	10	23.5	0.61	7.4	798	134.5

### Pyrosequencing of tagged amplicons and diversity of microbial community

Purified environmental DNA was used as a template for direct amplification of the partial 16S rRNA gene (0.44 kb) and ITS regions (0.5–1 kb) attached with specific tag sequences. The amplicons were sequenced and grouped according to their tags. The pyrosequencing dataset for filtered reads is summarized in Table 2. In total, 369,780 filtered reads were obtained with an average read length of 398.8 bases. Individual 16S rRNA gene samples contained 1,390–72,220 reads with the lowest number in BW1 and the highest in BW10. Fungal ITS sequence dataset (FS1–FS10 and FW1–FW10) contained 420–19,388 reads/sample with the lowest in FS10 and the highest in FW7. Little variation in %GC was observed among different samples.

**Table 2:** Summary of pyrosequencing dataset.

Bacterial 16s rRNA gene			Fungal ITS		
Sample ID	No. sequence	Average length	Sample ID	No. sequence	Average length
BS1	3,816	391.5	FS1	9,941	392.2
BS2	2,867	403.1	FS2	7,050	398.3
BS3	1,390	405.1	FS3	6,518	426.1
BS4	3,301	409.9	FS4	3,670	388.5
BS5	18,796	411.5	FS5	957	374.5
BS6	6,314	405.3	FS6	2,529	411.0
BS7	17,229	411.5	FS7	4,991	429.4
BS8	6,229	397.7	FS8	1,918	391.3
BS9	2,562	391.2	FS9	1,251	392.7
BS10	2,436	369.6	FS10	420	446.9
BW1	1,523	388.1	FW1	19,388	400.1
BW2	1,992	404.2	FW2	15,294	377.9
BW3	1,768	376.2	FW3	13,177	387.8
BW4	13,838	414.7	FW4	8,193	402.4
BW5	21,952	415.3	FW5	2,545	390.7
BW6	8,491	392.9	FW6	4,357	442.6
BW7	10,993	407.2	FW7	21,261	397.6
BW8	25,252	393.6	FW8	2,998	411.0
BW9	14,751	412.9	FW9	3,081	419.5
BW10	72,200	386.7	FW10	2,541	281.9

To identify unique phylotypes and estimate microbial community richness, the OTU in the samples was assigned from the cleaned sequences by the furthest-neighbor method of MOTHUR at 97% similarity levels. The results revealed that the bacterial phylotypes ranged from 108 to 522 OTUs per sample (Table 3). The highest and lowest species richness for the bacterial 16S rRNA gene were found in BS7 and BW1, respectively. This was in accordance with the Chao1 richness estimator. Shannon diversity index of bacterial diversity was higher in sediment (5.26–6.42) than water samples (4.25–5.87), indicating a more diverse bacterial community in sediments. Good’s coverage showed that the bacterial diversity obtained in this study covered a substantial fraction of bacterial species (68.54–92.23%) in different samples, and covered >74.88% of the estimated genera.

**Table 3:** Statistical analysis and biodiversity index of bacterial 16S rRNA gene and fungal ITS1 tagged-pyrosequences at the 97% similarity.

Bacterial 16s rRNA gene					Fungal ITS				
Sample ID	OTU	Chao1	Shannon	Good’s Coverage	Sample ID	OTU	Chao1	Shannon	Good’s Coverage
BS1	352	1,489	6.26	76.96	FS1	604	3,960	6.58	62.99
BS2	408	1,503	6.12	71.76	FS2	573	3,457	6.55	57.46
BS3	184	753	5.26	80.85	FS3	554	3,407	6.52	59.32
BS4	378	1,633	6.01	74.88	FS4	615	3,826	6.60	48.57
BS5	474	2,352	6.35	85.13	FS5	251	1,194	5.68	40.55
BS6	409	1,999	6.12	79.72	FS6	582	2,557	6.51	42.96
BS7	522	3,148	6.42	81.10	FS7	637	4,145	6.61	49.71
BS8	433	2,543	6.15	76.47	FS8	487	2,504	6.27	35.42
BS9	404	1,452	6.16	68.54	FS9	333	1,905	5.93	37.60
BS10	281	1,225	5.74	76.63	FS10	114	657	4.90	32.80
BW1	108	455	4.56	85.42	FW1	670	6,253	6.62	62.00
BW2	160	810	4.82	83.97	FW2	632	4,313	6.66	64.32
BW3	132	773	4.76	80.84	FW3	525	3,530	6.42	66.33
BW4	225	1,344	4.91	89.83	FW4	684	5,045	6.68	49.20
BW5	226	1,316	4.90	91.44	FW5	496	2,876	6.38	42.04
BW6	253	1,318	5.57	86.33	FW6	493	2,909	6.54	47.05
BW7	178	1,246	4.53	89.93	FW7	728	6,040	6.76	56.38
BW8	301	1,637	5.63	89.63	FW8	594	3,585	6.57	46.30
BW9	161	957	4.25	92.23	FW9	562	4,794	6.65	46.55
BW10	365	2,244	5.87	90.54	FW10	366	1,646	6.12	56.96

The phylotypes of ITS1 data represented 114 to 728 OTUs/sample, which was highest in sample FW7 and lowest in sample FS10 (Table 3). The Chao1 richness estimators of fungal diversity were highly varied among samples. The Shannon diversity index suggests variations in fungal diversity in different samples, ranging from 4.90 to 6.76 with slightly higher diversity in the water phase. The highest Shannon diversity index was found in FW7, while FW1-2 and FW8-10 also showed relatively high diversity compared with the sediment samples. The Good’s coverage indicated 32.80%–66.33% coverage of fungal species by the data set and 33.33–70.27% coverage of the estimated genera. The relatively low coverage could be due to the overestimation of OTUs resulting from the high sequence variability of ITS compared to 16S rRNA gene sequences [26].

### Phylogenetic analysis of flood microbial communities

Taxonomic classification of the bacterial 16S rRNA gene was performed based on the cleaned sequences using RDP classifier tool with 80% confidence threshold, which allowed >95% accuracy on identification at the genus level from the V3 and V4 regions [19]. In total, 43.06% of 16S rRNA gene sequences could be assigned to the genus level, and 21.61% to the family level, while the remainder could only be roughly assigned to higher taxonomic levels. A substantial fraction of 16S rRNA gene sequences, accounting for 9.99% of the total sequences, could not be classified into any known phyla based on the RDP classifier tool.

The taxonomic classification of fungal ITS was performed by BLAST against the custom ITS database [20]. For fungal diversity, a total of 98.60% ITS query sequences could be hit against the database with an average E-value of 1e-03. The majority (69.56%) of the total BLAST hit could be classified to the genus level. A significant fraction (9.50%) of fungal ITS1 sequences did not match any sequences in the database based on the cut-off threshold. Moreover, 1.75% of fungal ITS sequences showed a close similarity to unclassified fungi. Most were in the sediment samples, suggesting the unexplored nature of microbial diversity in the environments.

### Bacterial diversity

**Fig 2:**
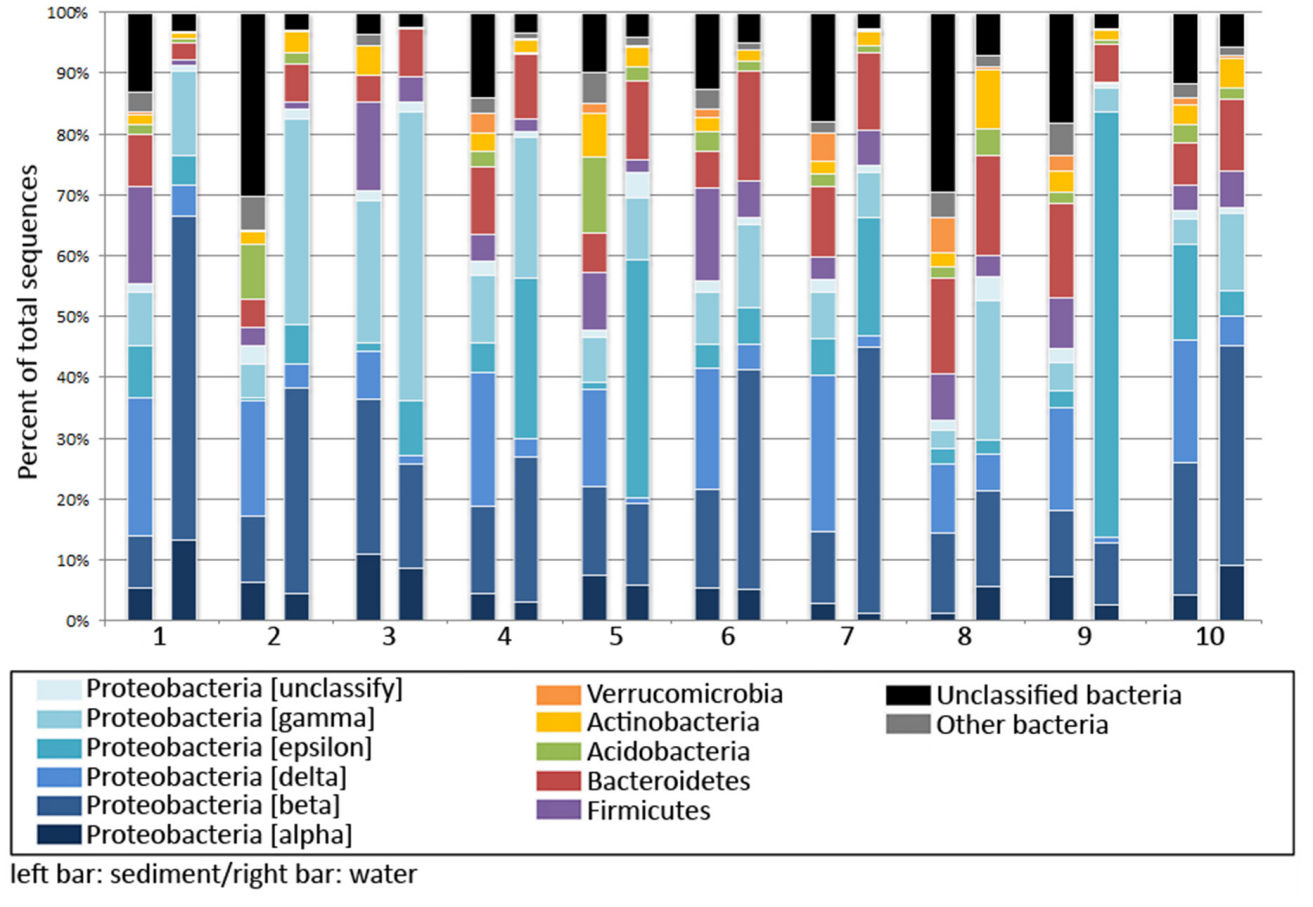
Comparison of bacterial diversity in sediment and water. Percent relative abundance of bacterial phyla based on 16S rRNA gene sequence abundance are shown. Left and right bars represent relative abundance collected from sediment and water, respectively.

Analysis of the pyrosequencing dataset revealed the overall differences in bacterial community structures in the sediment and floodwater samples (Fig 2). At the phylum level, the bacterial community structures in sediments showed overall similarities among different samples (BS1-BS10). Proteobacteria represented the most abundant phylum in all sediments (32.98%–70.71%), while Bacteroidetes, Firmicutes, and Acidobacteria also contributed substantial fractions to the communities. Compared to sediments, bacterial communities in the floodwater contained remarkably higher relative abundance of Proteobacteria (56.50%–91.40%), while Bacteroidetes represented the second most abundant phylum in all water samples. A significantly higher fraction of unclassified bacteria was found in the sediment samples (3.51%-30.25%) compared to the water samples (<7.12%), suggesting the less explored bacterial taxonomy associated with sediments.

**Fig 3:**
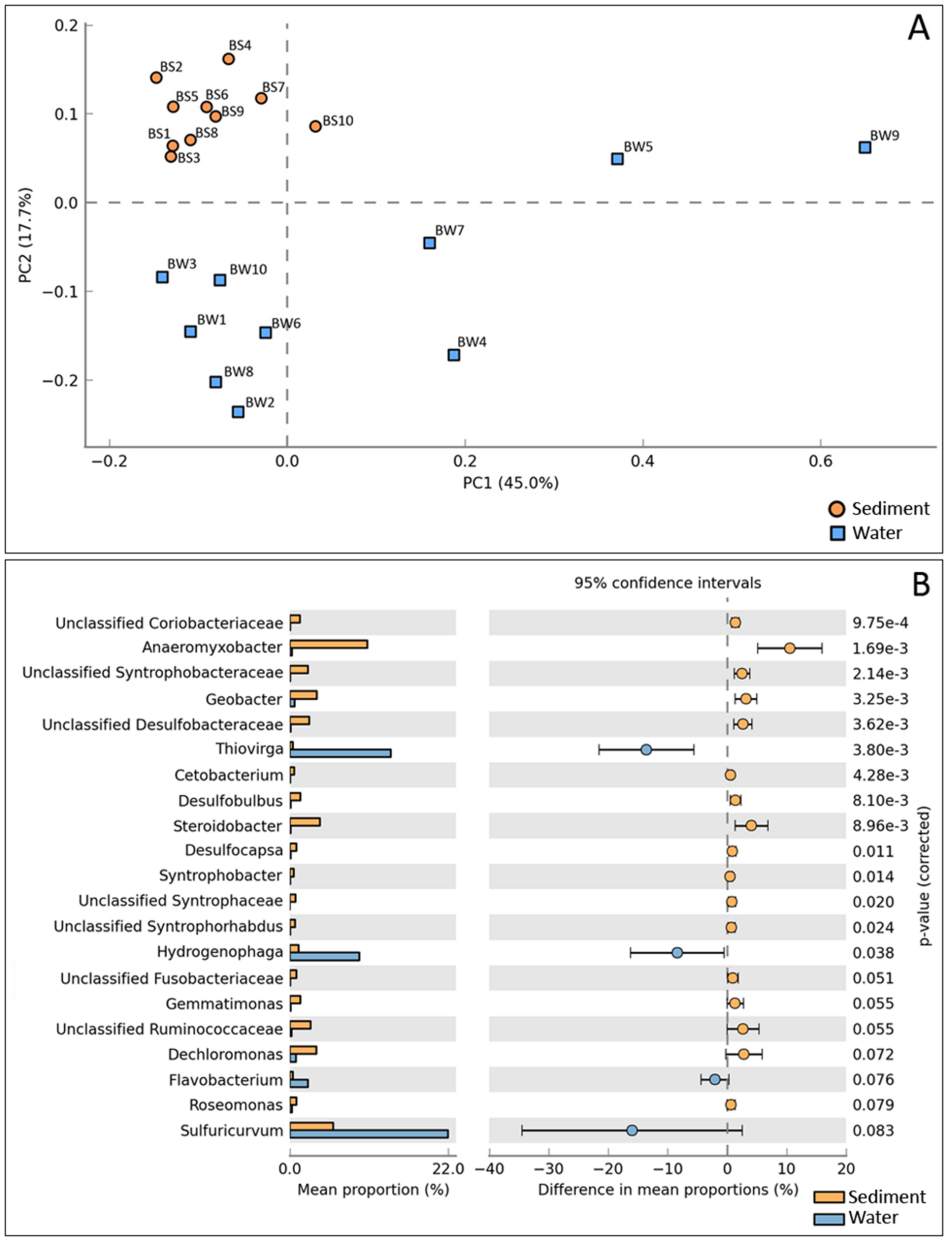
Comparative distribution of bacterial diversity in sediment and water. (A) Principal component analysis (PCA) based on percent relative abundance of bacterial taxa at genus level in sediment (orange) and water (blue). (B) Bar chart with extended error bar for pair-wise comparisons of bacterial genera between sediment (orange) and water (blue) samples.

A cluster analysis showed relationships of bacterial populations in different sediment and water samples based on quantitative taxa similarities at different taxonomic levels. According to the principal component analysis (PCA), all sediment samples shared highly similar common bacterial taxa at the phylum level while they tended to diverge and form distinct clusters at the deeper taxa levels (Fig 3A). For example, the diversity in all sediment samples were closely related, showing an overall apparent similarity in community structures, while BW4, BW5, BW7, and BW9 are distantly related to other bacterial communities in the water samples. No strong relationship was found between the bacterial profiles in the sediments and floodwaters from the same site as well as for any relatedness between samples from the agricultural and residential areas. The results clearly demonstrated heterogeneity of bacteria across different environments in the flooded areas.

At the deep taxonomic level, the bacterial diversity at different locations showed marked differences in bacterial genus profiles. A pairwise analysis showed a clear separation of key bacterial genera in the sediments and floodwaters. *Anaeromyxobacter*, *Steroidobacter*, *Dechloromonas*, and *Geobacter* represented the major genera in sediment samples from different locations. In contrast, *Sulfurivucum*, *Thiovirga*, *Hydrogenophaga*, and *Flavobacterium* represented the major bacterial taxa in floodwater samples (Fig 3B). In total, 21 genera showed significant differences in their distribution in sediment and water phases (*p* < 0.1), suggesting variations in the relative richness of individual genera. A comparative analysis of the twenty most abundant bacterial genera revealed differences in their diversity profiles between sediment and water from the same site (S1 Fig). *Anaeromyxobacter* was the most abundant genus in all sediment samples except in BS1, in which *Sulfurovum* was the major genus, and BS3, which was dominated by *Pseudomonas* and *Steroidobacter*. *Sulfuricuvum* existed as the dominant genus in four water samples while *Thiovirga* represented the most abundant genus in the other three water samples.

### Fungal diversity

**Fig 4:**
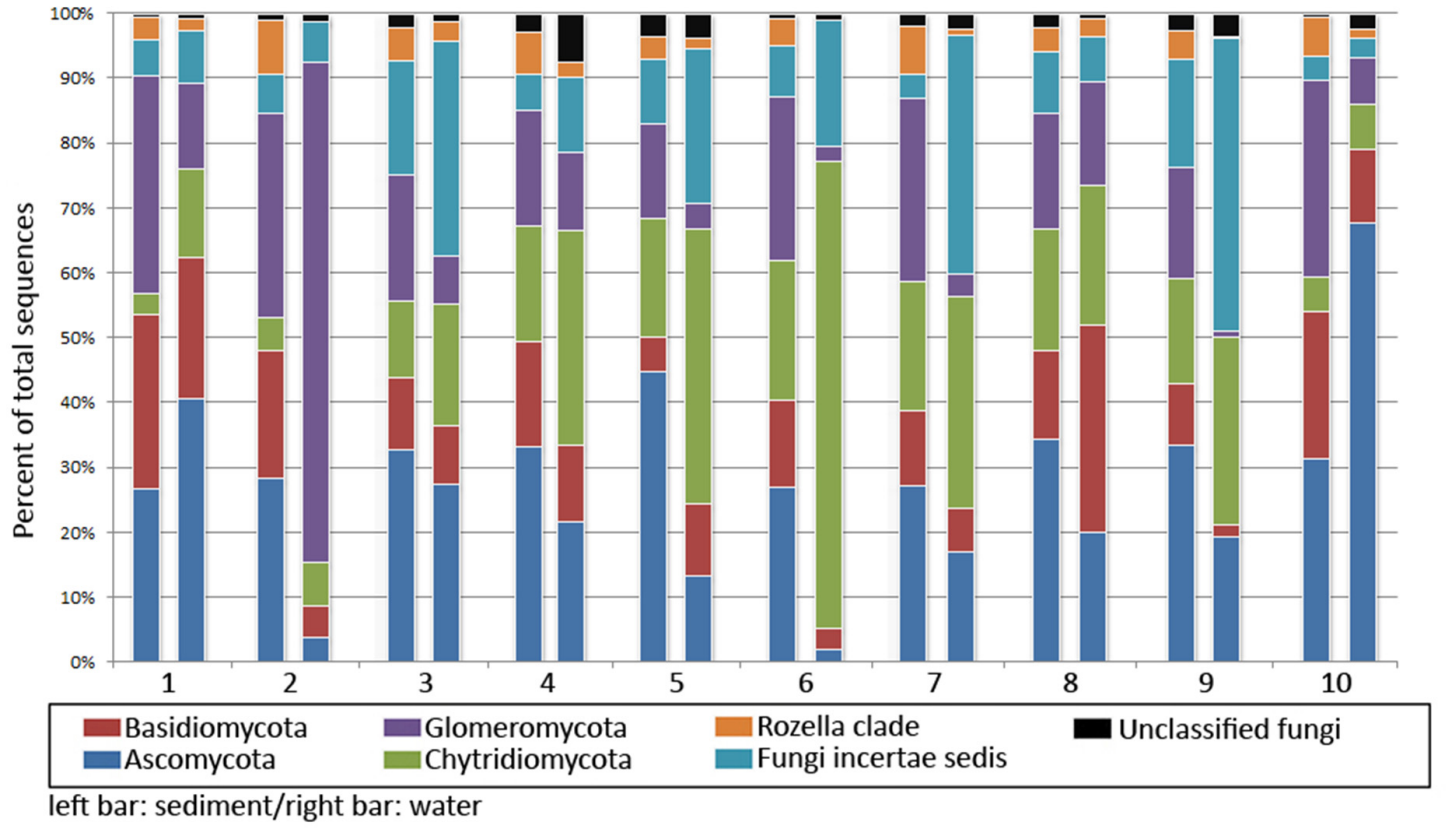
Comparison of fungal diversity in sediment and water. Percent relative abundance of fungal phyla based on ITS sequence abundance are shown. Left and right bars represent relative abundance collected from sediment and water, respectively.

Substantial differences were found among the fungal diversity from sediments and floodwater from different locations according to taxonomic assignment based on the ITS1 sequences (Fig 4). Ascomycota (1.92%–67.79%) and Glomeromycota (0.80%–77.04%) were the most abundant fungal phyla in sediment, accounting for >50% of sequences (FS1–FS10), while Basidiomycota and Chytridomycoda were minor phyla in all communities. Although they comprised similar composite phyla to those in the sediment samples, differences in their relative phyla abundance in the overall community structures were observed for the water samples. Ascomycota was the most abundant phyla in FW1 and, particularly, FW10, where it comprised nearly 70% of the total fungal diversity. Glomeromycota represented >75% of the total ITS sequences in FW2, and Chytridomycota accounted for the majority of fungal diversity in FW5 and FW6. A substantial fraction of Fungi incertaesedis was found for FW4, FW7, and FW9 where they contributed the largest fraction in the communities. Overall, similar fungal phyla profiles were obtained using taxonomic classification based on ITS4 (data not shown).

**Fig 5:**
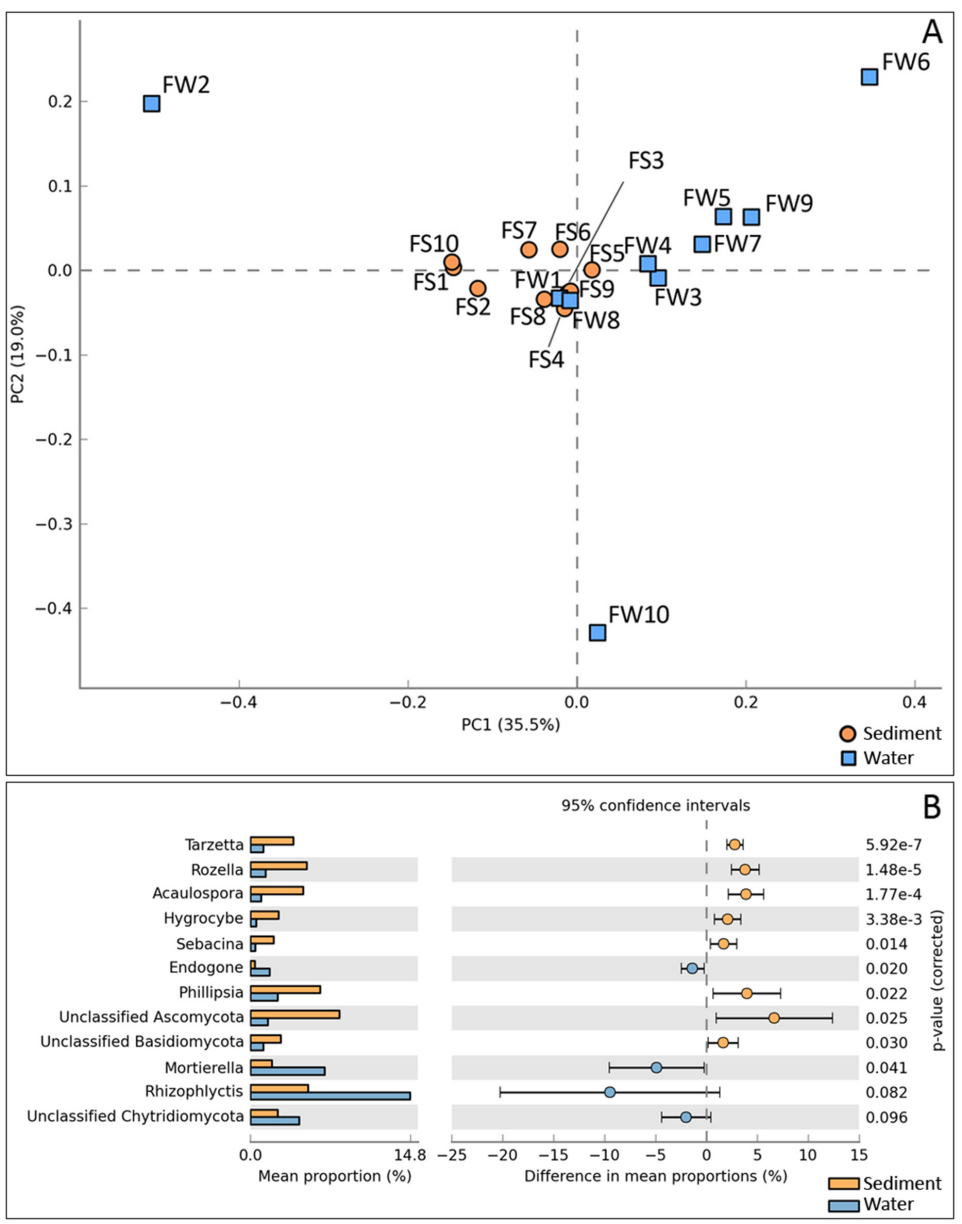
Comparative distribution of fungal diversity in sediment and water. (A) Principal component analysis (PCA) based on percent relative abundance of fungal taxa at genus level in sediment (orange) and water (blue). (B) Bar chart with extended error bar for pair-wise comparisons of fungal genera between sediment (orange) and water (blue) samples.

Correlation analysis by Principle Component Analysis (PCA) showed that most communities in sediments and waters shared significant similarities in overall diversity at the genus level. However, FW2, FW6, and FW10 were the exceptions (Fig 5A). At the deep taxonomic levels, fungal communities at various locations showed marked differences in the genus profiles. A pair-wise analysis showed a distinct separation of dominant fungal genera in the sediments and floodwaters. Genera *Tarzetta*, *Philipsia*, *Rozella*, and *Acaulospora* as well as unclassified *Archaeosporales* and unclassified Ascomycota represented the major genera in the sediments. In contrast, *Rhizophylctis*, *Mortierella*, *Endogone*, and unclassified Chytridomycoda represented the major fungal taxa in the floodwater from different sites (Fig 5B). A comparative analysis of the twenty most abundant fungal genera in the sediment and water samples revealed differences in their diversity profiles (S2 Fig). Unclassified Archeosporales represented the major genus in all sediment samples as well as in two water samples (FW1 and FW2). *Rhizophlyctis* was the major genus in FW3, 4, 5, 6, and 7.

### Analysis of sulfate-reducing bacteria diversity

A further investigation examined the diversity of sulfate-reducing bacteria using DGGE analysis of the *dsr*B gene amplicons in selected samples. The *dsrB* gene could be amplified only from the sediment samples (BS1, BS2, BS3, and BS5) but not from the water. Amplicons from the 4 positive sediment samples showed different band patterns on DGGE gels, indicating variations in sulfate-reducing bacterial communities among the samples.

**Fig 6:**
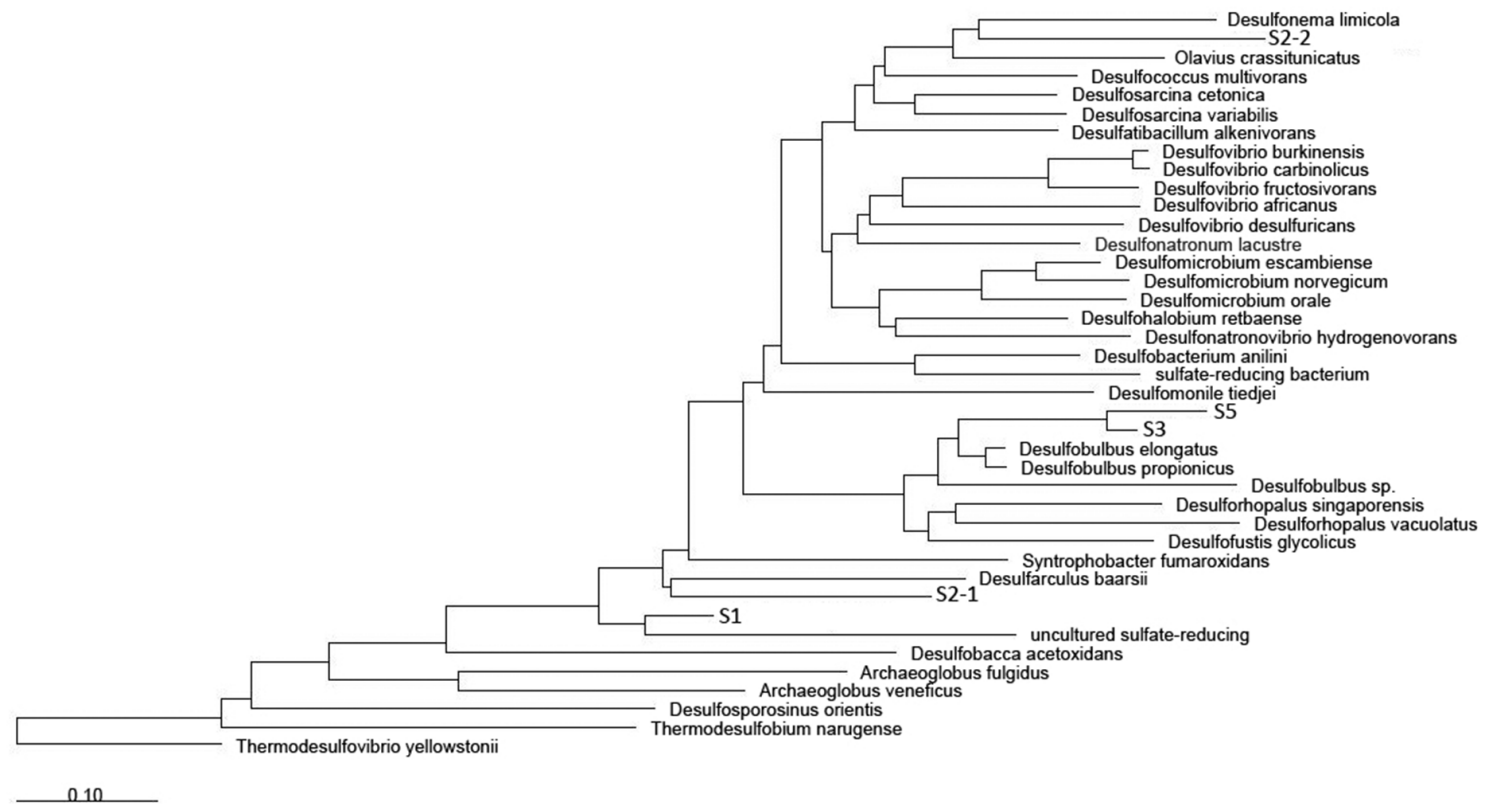
Phylogenetic tree of *dsrB* genes from DGGE analysis of sulfate-reducing bacteria.

Fig 6 shows a phylogenetic tree of *drs*B genes from sediment samples. Sulfate-reducing bacteria related to families Desulfobacteraceae, Desulfobulbaceae, Syntrophobacteraceae (genus *Desulfomonile*), and Desulfoarculaceae (genus *Desulforaculus*) as well as one uncultured sulfate-reducing bacterium were found in the samples.

## Discussion

The flood ecosystem is considered a temporally variable ecological-niche within the aquatic environment that can have a major impact on microbial communities related to water quality and public health. Compared to marine ecosystems, microbial ecology in freshwater aquatic systems has received little attention, despite the greater potential to negatively impact humans, both on physical damage of habitats and public health concerns. Analysis of microbial diversity in aquatic systems, such as fresh water sediments in lakes and periodically flooded plains, has been conducted using culture-independent molecular methods (clonal library analysis and fluorescence in situ hybridization) [27, 28]. Recent meta-analyses revealed higher abundance of microbial taxa in inland freshwater than in marine environments [7]. Presence of pathogenic bacteria in floodwater and consumption water during the Thailand’s flood crisis has been reported by detection of specific marker genes [4]. However, no in-depth study on microbial community in floodwater has been conducted with only few exceptions on surveying microbial diversity by isolation techniques [3] and 16S rDNA clonal libraries [29].

Physiochemical analysis of floodwater samples in this study showed varying DO, reflecting varying oxygen levels from aerobic to anaerobic conditions. The observed wide range of redox conditions could support various groups of microorganisms, possessing different metabolic capabilities. Biogeochemical cycles during flooding are related to diversity of microorganisms, which function interactively in various biochemical activities, such as decomposition of organic matter by saprophytes, mineralization by oxidizers, denitrification/sulfate reduction by denitrifiers/sulfate reducing bacteria and methanogenesis by archaea. Bacteria and fungi are major decomposers of organic matter in aquatic systems and play key roles in nutrient cycling, mineralization and other biochemical activities. Their interaction and overlapped activity in organic matter decomposition in aquatic environments have been shown [30, 31]. These microbial processes are directly related to the physical and chemical qualities of floodwater which affect human habitats and health in the flood area [2].

Bacteria-to-fungi ratios correlated with key physicochemical parameters such as organic carbon, nitrogen, and pH [32]. According to our study, sediment and water supported different lineages of dominant bacteria and fungi. Diversity indices showed a higher diversity of bacteria in sediments than in water bodies, in contrast to the slightly higher fungal diversity in water, suggesting differences in the microbial processes in these phases. In the flood ecosystem, sediment, resulting from the combination of organic matter deposited from the upper water layer and organic matter already present on the ground, provides a nutrient matrix to support microbial growth and a wide range of biochemical activities. The high content of organic matter and nutrients in fresh water sediments can support greater microbial growth and lead to intense mineralization. Diverse bacteria and fungi have been recorded in sediments in an aquatic system using illumina tag sequencing [7]. In contrast to sediment, the water phase is characterized as a suspension of insoluble organic substances and soluble organic matter. The water community is more temporally unstable due to its flows, particularly during the first stage of flooding before it changed to the waterlogged condition afterwards.

Various groups of microbes were detected in the flood ecosystems, including primary producers, saprophytic microbes, and those involved in sulfate reduction under anaerobic conditions. The result showed that distinct microbial phyla are enriched in sediments and waters. *Proteobacteria*, the largest bacterial phylum with versatile metabolic capabilities, was the predominant bacterial group in sediments and waters. It has been suggested that the predominance of *Alphaproteobacteria* and *Betaproteobacteria* in freshwater sediments relates to pH and nutrients in the environment [7]. *Gammaproteobacteria* and *Epsilonproteobacteria* are enriched in marine sediment and intertidal wetland, respectively, while *Deltaproteobacteria* are present in sediments from freshwater and marine environments [7]. The majority of the *Gamma-* and *Deltaproteobacteria* in aquatic sediments were sulfur reducers. The presence of complex organic matters in flood sediment and water also supported the enrichment of primary consumers and saprophytic microbes such as *Bacteroidetes*, *Firmicutes*, and *Actinobacteria*, which collectively constituted a complex ecological niche in the flood ecosystem. These various groups of bacteria are expected to conduct decomposition of organic matter in sediment and water during flooding under the conditions with varying oxygen availability. The finding implies the complex ecological microbial processes starting from degradation of complex organic compounds to subsequent metabolisms related to water quality in the ecosystem.

At the genus level, *Sulfuricurvum*, *Thiovirga*, *Hydrogenophaga*, *Tolumonas*, *Flavobacterium*, and *Novosphingobium* were among the most enriched bacterial taxa in water. Many of these microbial taxa have been previously isolated from aquatic or marine environments. The most abundant taxon, the facultative anaerobic and chemolithoautotrophic *Sulfurivurum*, is a member of Epsilonproteobacteria that is capable of sulfur lithotrophy [33]. This group of sulfur oxidizers was found in aquifers and oil contaminated sites [34, 35]. *Sulfuricuvum* and *Hydrogenophaga*, responsible for nitrate reduction, and *Dechloromonas*, performing perchlorate reduction in biofilm, have been reported to coexist in a hydrogen-based membrane reactor [36]. A Betaproteobacteria genus, *Hydrogenophaga*, played an important role in the decomposition of organic matter in wastewater in a biofilm bioreactor designed for chemical oxygen demand removal [37]. *Hydrogenophaga* are versatile aerobic bacteria capable of degrading polluted hydrocarbons including benzene, methyl-*tert*-butyl ether, and polycyclic aromatic hydrocarbons (PAHs) [38, 39]. *Thiovirga* is a group of sulfur oxidizing bacteria that has been found in landfill cover soil together with other sulfur oxidizing and sulfate reducing bacteria involved in sulfur bioconversion [40]. *Tolumonas* is a facultative anaerobic Gammaproteobacteria, which was previously isolated from wastewater and anoxic sediment of a fresh water lake [41, 42]. It produces ethanol and short-chain organic acids from glucose metabolism. *Novosphingobium* is a group of nitrogen fixing bacteria capable of hydrocarbon degradation. *Novosphingobium* strains were previously isolated from mangrove sediments [43] and oil-affected wetland and seawater samples [44]. This dominant microbial taxa profile suggested predominance of mixed anaerobic and aerobic metabolism related to decomposition of organic matter and xenobiotic compounds in the water samples studied.

In contrast to the water phase, *Anaeromyxobacte*r, *Dechloromonas*, *Geobacter* and *Steroidobacter* were the most dominant bacterial genera in sediment. The majority of these genera are anaerobic or facultatively anaerobic bacteria that reside in anoxic environments. Most are involved in reduction of sulfates/nitrates and degradation of xenobiotic compounds [45–47]. *Anaeromyxobacteris*, a metabolically versatile Deltaproteobacteria, which has a bioremediation capability, was found in flooded paddy soil using PCR-RFLP and real-time PCR of 16S rRNA gene [48]. This group of bacteria can utilize a wide variety of electron acceptors for growth under anoxic conditions, including ortho-substituted halophenols, nitrate, oxygen, fumarate, and Fe(III). *Anaeromyxobacter*-related species and *Geobacter* are important metal reducing bacteria in acidic subsurface sediments [49]. Strains of the Betaproteobacteria genus *Dechloromonas* have been reported to degrade various hydrocarbons in groundwater [50]. They also are capable of chlorate, nitrate, and iron reduction in wetland environments [51, 52]. Its coexistence with other sulfate reducing bacteria in contaminated water sediments e.g. *Geobacter* which are potent sulfate and iron reducers capable of anaerobic degradation of aromatic hydrocarbons [53] in an enriched nitrate reducing and Fe(III)-reducing microcosm originated from microflora in flood plain has also been shown [54, 55]. In addition, *Flavobacterium* is a large bacterial genus comprising diverse members capable of degrading xenobiotic compounds such as phenol [56]. A *Steroidobacter denitrificans* strain was isolated from microbial community capable of ammonia/nitrate removal in a constructed wetland with waste water influent [57]. Overall the bacterial diversity suggested complex biochemical processes involving degradation of organic and xenobiotic compounds as well as in oxidoreductive nitrate/sulfate cycling in the sediment phase.

Odor problem in the flood areas is also considered an important public health concern. In general, unpleasant odor is associated with the decomposition of organic matters, especially under anaerobic conditions [58]. This involves generation of volatile fatty acid, ammonia and hydrogen sulfide by various biochemical processes e.g. fermentation of organic compounds, protein degradation and sulfate reduction [59]. Microbial communities of sulfate-reducing bacteria, which is a key microbe related in odor generation were further investigated based on diversity of the *dsr*B genes. Deltaproteobacteria related to Desulfobacteraceae, *Dsesulfomonile tiedjei*, Desulfobulbaceae, Syntrobacteraceae, and Desulfoarculus were found to be key sulfate-reducers in the flood environment. Their roles on organic matter recycling under anaerobic conditions have been reported [60–64]. The presence of several genera in family Desulfobacteraceae and Desulfobulbaceae was also found in the pyrosequencing dataset of both sediment and water. In addition, a number of photosynthetic purple non-sulfur bacterial strains were isolated from several flood samples in this study (data not shown). Most were closely related to *Rhodopseudomonas palustris*, based on analysis of 16S rRNA gene and the specific *puf*M gene, which encodes a protein for the M subunit of the photosynthetic reaction center. The roles of these purple nonsulfur bacteria in cooperative actions with sulfate-reducing bacteria have been reported [65].

In addition to bacteria, fungi contribute to organic matter decomposition in aquatic ecosystems [32]. Ascomycota, Basidiomycota, Chytridiomycota were predominant fungal phyla that were widely distributed in floodwater and sediment. The saprophytic roles of these fungi related to the decomposition of organic matter in aquatic environments have been reported [30, 66]. Ascomycota is a large fungal phylum comprising both yeasts and filamentous fungi. Their functions in the degradation of various organic substrates in soil and water are well documented. Basidiomycota decomposed organic matter, particularly lignocelluloses, in soil and aquatic ecosystems. The role of Chytridiomycota in organic matter degradation in an aquatic ecosystem has been reported [32]. Increasing relative abundance of Chytridiomycota in soil fungal communities was found to correlate with increasing extracellular enzymatic activities on cellulose and hemicellulose in soil [67], while they also play a role on organic matter decomposition in aquatic ecosystems [68].

*Rhizophlyctis*, *Mortierella*, unclassified *Chytridiomycota*, *Aspergillus*, and *Rhizopus* are the most dominant fungal genera in water. *Aspergillus* and *Rhizopus* are well-known degraders of organic compounds in various environments, including aquatic ecosystems. They are capable of producing a variety of hydrolytic enzymes targeting decomposition of plant polysaccharides, protein, and lipid, and are key decomposers in nutrient cycling under aerobic conditions. The dominance of *Rhizophlyctis* in a PAH degrading fungal community and their ability to degrade cellulose has been reported previously [69]. *Motierella*, oleaginous fungi that accumulate fatty acid [70], are frequently isolated from soil where they decompose organic matter saprophytically [66]. In contrast to the fungal diversity in water, unclassified *Ascomycota*, *Philipsia*, *Rozella*, and *Acaulospora* predominated in sediment. *Rozella* has been reported in marine environments but not in aquatic ecosystems [71]. With relatively limited data on these fungi, a further study is needed to give insights into the roles of fungal diversity in the water bodies.

In conclusion, an extensive survey of microbial diversity in temporally variable flood ecological niches has been firstly reported in our work using the high-throughput tagged amplicon pyrosequencing technique. Differences in bacterial and fungal taxa distribution in the sediment and water indicated different dominated biochemical activities and microbial processes in these phases. This diversity survey provides a foundation for more detailed studies on how microbial communities in flood ecosystems affect physicochemical changes in the environment and impact human communities.

## Supporting Information

S1 Fig Heat-map of key bacterial taxa at genus level. The color ranges represent the relative abundance of bacteria collected from different locations: sediment (left); water (right).
(TIF)

S2 Fig Heat-map of key fungal taxa at genus level. The color ranges represent the relative abundance of fungi collected from different locations: sediment (left); water (right).
(TIF)
