# Supplementary figures and images for: Survey of Microbial Diversity in Flood Areas during Thailand 2011 Flood Crisis Using High-Throughput Tagged Amplicon Pyrosequencing
Source: PLoS One. 2015 May 28;10(5):e0128043. doi: 10.1371/journal.pone.0128043 (PMC4447364; doi:10.1371/journal.pone.0128043)

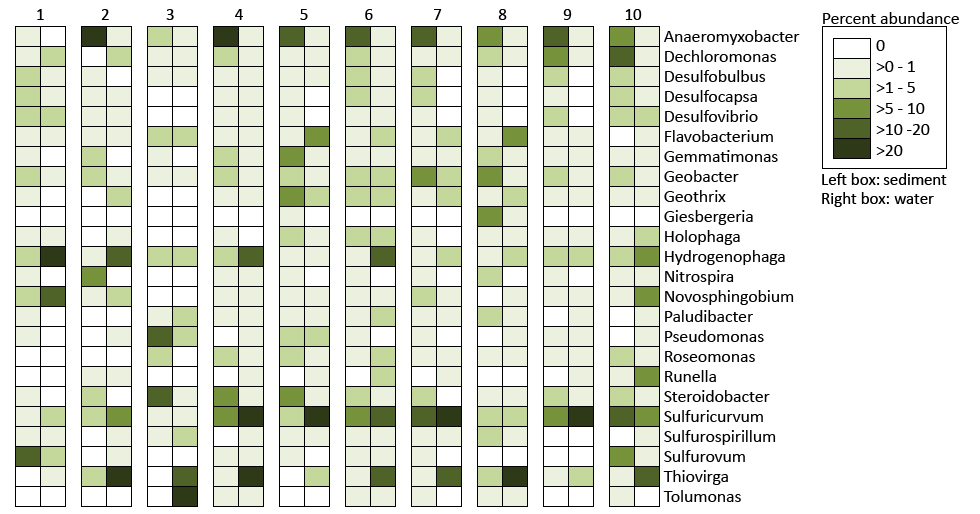

Supplement: S1 Fig — The color ranges represent the relative abundance of bacteria collected from different locations: sediment (left); water (right). (TIF) [file pone.0128043.s001.tif]

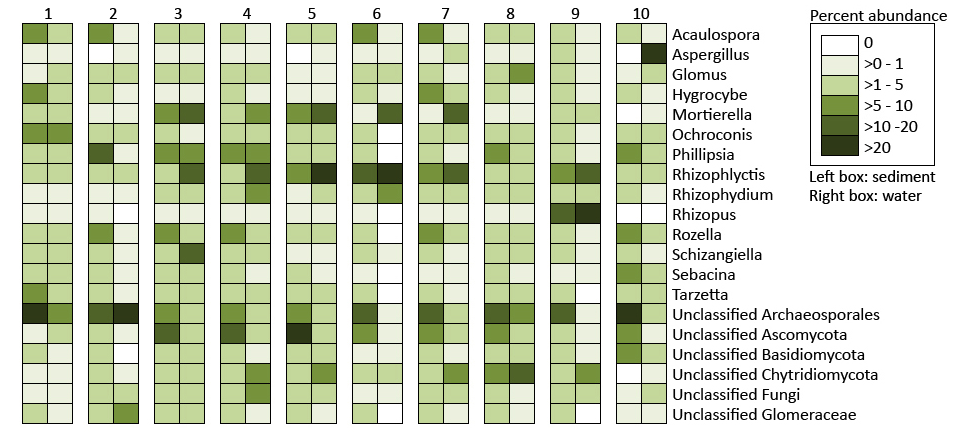

Supplement: S2 Fig — The color ranges represent the relative abundance of fungi collected from different locations: sediment (left); water (right). (TIF) [file pone.0128043.s002.tif]
